# 25-hydroxyvitamin D and testosterone levels association through body mass index: A cross-sectional study of young men with obesity

**DOI:** 10.3389/fendo.2022.960222

**Published:** 2022-09-02

**Authors:** Miguel Damas-Fuentes, Hatim Boughanem, María Molina-Vega, Francisco J. Tinahones, José C. Fernández-García, Manuel Macías-González

**Affiliations:** ^1^ Department of Endocrinology and Nutrition, Virgen de la Victoria University Hospital, Institute of Biomedical Research in Malaga (IBIMA), University of Malaga, Malaga, Spain; ^2^ Spanish Biomedical Research Center in Physiopathology of Obesity and Nutrition (CIBERObn), Instituto de Salud Carlos III , Madrid, Spain

**Keywords:** vitamin D, obesity, morbid obesity, total testosterone, sex-related hormone

## Abstract

**Backgrounds:**

Vitamin D and testosterone deficiency have been widely related to obesity. However, only a few studies have investigated the effect of vitamin D on testosterone in the context of obesity, in which controversial results have been raised.

**Objectives:**

The purpose of this study was to determine the relationship between serum 25-hydroxyvitamin D (25(OH)D) and testosterone levels in young men with different grade of obesity.

**Design and methods:**

This cross-sectional study included 269 healthy young men with obesity (body mass index (BMI) ≥ 30 kg/m^2^). Participants were divided into two groups based on their serum 25(OH)D levels (134 subjects with vitamin D sufficiency and 135 participants with vitamin D deficiency, according to the 50^th^ percentile of 25(OH)D). Serum 25(OH)D and sex hormones have been measured. The relationships between 25(OH)D, sex hormones, and obesity grades were investigated with linear and binary logistic regression analyses, as well as mediation analysis.

**Results:**

Compared to the 25(OH)D sufficiency group, total and free testosterone levels were found to be decreased, whereas serum androstenedione levels were increased in the 25(OH)D deficiency group (*p*<0.05). Using multivariable lineal regression analyses, 25(OH)D was correlated with the majority of sex hormones (*p*<0.05). When mediation with BMI was performed, the direct effect between 25(OH)D and sex hormones disappeared, and only the indirect effect *via* BMI remained (demonstrating the importance of BMI). Furthermore, after controlling for age and smoking status, we discovered that total testosterone and SHBG were both significantly associated with 25(OH)D (*p*<0.05) in subjects with obesity type III. Using a mediation analysis, we discovered that BMI had a partial effect on the association between 25(OH)D and total testosterone levels in morbidly obese participants, indicating that a direct association between 25(OH)D and total testosterone levels, and that BMI partially mediated this association.

**Conclusions:**

Serum 25(OH)D is associated with total testosterone levels in only those subjects with morbid obesity, suggesting a specific benefit in severe cases of obesity. Additional research is needed to elucidate possible common mechanisms.

## Introduction

Apart from the global dimensions of obesity, which is already the biggest problem for public health, obesity is one of the factors that negatively influences human health (1). The causes of obesity are complex, which makes it difficult to pinpoint exactly why the worldwide prevalence of obesity has nearly tripled in the last 40 years (2). The urgency of tackling obesity has been brought to the fore by evidence of a link to an increased risk of several metabolic diseases/disorders, such as type 2 diabetes, cardiovascular diseases, metabolic syndrome, or infertility, among others (3). Nonetheless, one of the most common observations in subjects with obesity, is the high prevalence of vitamin D deficiency (4). This relationship has been well-documented in numerous epidemiological studies, meta-analyses, and systematic reviews. Accordingly, a recent meta-analysis conducted by Fiamenghi et al. (2021) pointed out that overall and abdominal obesity are strong risk factors for vitamin D deficiency in both children and adolescents (5), but also in adults (6, 7). Moreover, a recent meta-analysis and randomized controlled trials observed that vitamin D supplementation in subjects with obesity did not have any effect on body mass index (BMI) nor percentage of fat mass (8, 9), suggesting a complex link.

On the other hand, obesity was largely related to an increased risk of low levels of testosterone in males, and this observation was consistently confirmed by several observational studies (10, 11). Additionally, testosterone supplementation/replacement in men with obesity had a positive effect on body composition and BMI (12, 13), indicating a close connection between adiposity and testosterone levels. Low serum 25-hydroxyvitamin D (25(OH)D) (the circulating form of vitamin D) levels have also been linked to low testosterone levels. Consequently, a recent systematic review and meta-analysis conducted by D’Andrea et al. (2021) reported a positive association between 25(OH)D and total testosterone levels (14). Additionally, a recent study found that vitamin D deficiency leads to reduced production of testosterone (15), which can affect male fertility, although there are some controversial results about this association (16). Thus, the association between vitamin D and testosterone levels is of clinical importance.

Nevertheless, as of today, only a few studies have investigated the effect of vitamin D on testosterone levels in the context of obesity, despite the previously mentioned data that strongly suggested a feasible relationship. Therefore, the aim of the present study was to investigate the relationship between serum 25(OH)D and testosterone levels in young men with different grades of obesity. We hypothesized that a low serum 25(OH)D levels in obese men would be associated with lower testosterone levels. Furthermore, we hypothesized that 25(OH)D levels would differ significantly across obesity grades, with variations of BMI having some influence on the relationship between vitamin D and testosterone levels.

## Material and methods

### Selection of participants

This cross-sectional study included 269 healthy young men with obesity (BMI ≥ 30 kg/m^2^) aged between 18 and 49 years old. The recruitment was conducted at six primary care centers in Málaga (Spain), between June 2013 and June 2015. Patients previously diagnosed by medical specialists with diabetes mellitus, hepatic or renal diseases, cardiovascular diseases, cancer, or other metabolic disorders were excluded from the study. Patients who had hypogonadism, or had received antidiabetic, hypogonadism or some treatments that alter serum 25(OH)D levels or activate the vitamin D receptor (VDR) were also excluded. Treatments targeted at the gonadal axis or testosterone levels were also excluded. All subjects had a normal pubertal development and an intact sense of smell. This study was reviewed and approved by the Ethics and Research Committee of Virgen de la Victoria Clinical University Hospital (Málaga, Spain) (Registration number CMCS240281), and was conducted according to the principles of the Declaration of Helsinki. Written consent has been obtained from each patient or subject after a full explanation of the purpose and nature of all procedures used.

### Biochemical determination

Blood samples were collected from all participants after fasting overnight. Serum samples were obtained by centrifugation for 15 min at 1800×g at 4°C. Serum glucose, total cholesterol, triglycerides, and high-density lipoprotein cholesterol (HDL-c) concentrations were determined by means of a Dimension Autoanalyzer (Dade Behring Inc., Deerfield, IL, USA). Low-density lipoprotein cholesterol (LDL-c) was calculated using the Friedewald equation (17). Insulin levels were measured by a radioimmunoassay method using BioSource International Inc. (Camarillo, CA, USA). We used Homeostasis Model Assessment Insulin Resistance Index (HOMA-IR), as described by Matthews et al. (1985), in order to determine the status of insulin resistance, using the following equation: HOMA-IR = fasting insulin (μIU/mL) × fasting glucose (mM)/22.5 (18).

### 25-hydroxyvitamin D and sex-related hormone determinations

An Enzyme-Linked ImmunoAssay (ELISA) kit (Immundiagnostik, Bensheim, Germany) was used to measure serum 25(OH)D levels. Total testosterone was determined by high-performance liquid chromatography mass spectrometry (HPLC-MS) (19). Sex-hormone-binding globulin (SHBG) was analyzed using an electrochemiluminescence immunoassay (Elecsys SHBG, Roche Diagnostics, USA). Free testosterone was calculated from total testosterone and SHBG using a law-of-mass-action equation (18). Serum insulin levels were measured by immunoassay using an ADVIA Centaur autoanalyzer (Siemens Healthineers, Erlangen, Germany). LH was determined by direct chemiluminometric assay (ADVIA Centaur; Siemens Healthineers, Erlangen, Germany); Follicle Stimulating Hormone (FSH), prolactin, dehydroepiandrosterone sulfate (DHEAs) and estradiol were measured using a direct chemiluminescent immunoassay (Siemens Healthineers Atellica IM 1600 Analyzer). Androstenedione was measured by an ELISA kit (Diagnostics Biochem Canada, Canada). The measurements of progesterone levels were conducted by an ELISA kit (IBL International GmbH, Germany). 17-OH-hydroxyprogesterone was determined by HPLC-MS conducted on a triple quadrupole liquid chromatography mass spectrometry system (Model 6460; Agilent Technologies, Santa Clara, California).

### Statistical analysis

Continuous variables are presented as mean values (standard deviation), and categorical variables are expressed as percentages. Baseline characteristics between groups were determined using Welch’s two sample tests for normal variables or the Mann-Whitney U for non-normal variables and a χ² test or Fisher’s exact test for categorical variables. To determine the relationship between sex hormones, 25(OH)D and obesity, as well as the influence of 25(OH)D on different grades obesity, linear, multiple regression and interaction analyses were performed (*p*<0.05). Furthermore, as an indirect effect, a mediated effect was tested to set if the effect of 25(OH)D on testosterone is mediated by BMI. The total effect of 25(OH)D was the sum of the direct (testosterone levels and BMI) and indirect (25(OH)D+BMI versus testosterone levels) effects. The significance of the mediation effect was performed using 5000 bootstrapped iterations mean indirect and direct effect. Data analyses were performed using the R v3.5.1. software (Integrated Development for R. RStudio, PBC, Boston, MA, USA), and significance was set at p<0.05 unless otherwise stated (20).

## Results

### Baseline characteristics of the participants according to the serum 25-hydroxyvitamin D

The baseline characteristics of participants are summarized in **Table 1**. The Participants were divided into two groups based on their serum 25(OH)D levels, in with 134 participants had normal/high serum 25(OH)D levels (vitamin D sufficiency) and 135 participants had low 25(OH)D levels (vitamin D deficiency) (50^th^ percentile). The value of the 50^th^ percentile of 25-hydroxyvitamin D fell over the value of 20.10 ng/mL.

**Table 1:** Summary descriptive table of population study, divided by the 50^th^ percentile of serum 25-hydroxyvitamin D.

Variables	25(OH)D sufficiency	25(OH)D deficiency	*p* value
N	N = 134	N = 135	
Age (years)	38.3 (7.58)	36.3 (8.05)	0.036*
Smoking (N (%))			0.431
No	54 (43.5)	62 (51.2)	
Past	35 (28.2)	32 (26.4)	
Current	35 (28.2)	27 (22.3)	
Weight (kg)	115 (16.1)	125 (22.6)	<0.001*
Waist circumference (cm)	120 (12.8)	128 (16.1)	<0.001*
Body mass index (kg/m^2^)	37.2 (5.61)	40.8 (7.32)	<0.001*
Free fat mass (%)	66.7 (5.46)	63.2 (5.98)	<0.001*
Fat mass (%)	32.7 (5.52)	36.3 (6.01)	<0.001*
Systolic blood pressure (mmHg)	132 (12.8)	133 (12.1)	0.635
Diastolic blood pressure (mmHg)	85.4 (10.00)	85.8 (8.64)	0.728
Glucose (mg/dL)	92.9 (11.6)	93.3 (10.3)	0.748
Insulin (μUI/mL)	18.4 (14.1)	21.7 (16.6)	0.080
Total cholesterol (mg/dL)	187 (35.0)	186 (32.8)	0.826
Triglycerides (mg/dL)	149 (81.3)	158 (78.0)	0.399
HDL-c (mg/dL)	42.2 (9.48)	41.9 (9.16)	0.799
LDL-c (mg/dL)	115 (30.4)	114 (28.5)	0.696
HOMA-IR	4.35 (3.72)	5.17 (4.79)	0.118
HbA1c (%)	5.42 (0.43)	5.42 (0.36)	0.931
25-hydroxyvitamin D (ng/mL)	27.3 (6.49)	14.2 (4.01)	<0.001*

25(OH)D sufficiency and deficiency groups are divided under the 50^th^ percentile of 25(OH). Data is expressed as mean ± standard deviations or n (percentage). Asterisk indicates significant difference between 25(OH)D sufficiency and deficiency groups, according to Mann-Whitney U test (*p < 0.05). Chi squared test was used for variables expressed as percentage (*p < 0.05).

25(OH)D, 25-hydroxyvitamin D: HbA1c, glycated hemoglobin; HOMA-IR, Homeostasis model assessment of insulin resistance; HDL-c, High-density lipoprotein cholesterol; LDL-c, Low-density lipoprotein cholesterol.

When comparing the 25(OH)D sufficiency and deficiency groups, we found significant differences in age.

The age of the 25(OH)D deficiency group was significantly lower than the age of the 25(OH)D sufficiency group (*p*<0.05). In addition, the 25(OH)D deficiency group had increased anthropometric variables, such as weight, waist circumference, BMI, and fat mass, when compared to the 25(OH)D sufficiency group (*p*<0.05). The serum 25(OH)D levels in the 25(OH)D deficiency group were significantly lower than those in the 25(OH)D sufficiency group (*p*<0.05), with the value of 25(OH)D (standard deviation), 14.2 (4.01) ng/mL, and 27.3 (6.49) ng/mL, respectively (**Table 1**).

### Sex-related hormone profiles according to the serum 25-hydroxyvitamin D

**Table 2** summarizes the sex-related hormone profiles, according to the 50^th^ percentile of 25(OH)D. We found that the 25(OH)D deficiency group had significantly lower serum total and free testosterone levels, than the 25(OH)D sufficiency group (*p*<0.05). In contrast, the 25(OH)D deficiency group had significantly increased serum androstenedione levels, in comparison with the 25(OH)D sufficiency group (*p*<0.05). Furthermore, when compared to the 25(OH)D deficiency group, the 25(OH)D deficiency group had a tendency (but non-significant; *p*=0.050) to decrease LH levels.

**Table 2:** Summary descriptive table of sex-related hormone profile in the population study, divided by the 50^th^ percentile of serum 25-hydroxyvitamin D.

Variables	25(OH)D sufficiency	25(OH)D deficiency	*p* value
N	N = 134	N = 135	
Total testosterone (ng/mL)	4.03 (1.47)	3.53 (1.30)	0.003*
Free testosterone (pg/mL)	94.9 (32.9)	85.4 (28.4)	0.012*
SHBG (nmol/L)	27.1 (13.9)	24.7 (11.3)	0.123
FSH (pg/mL)	4.25 (2.80)	3.70 (2.12)	0.074
Prolactin (ng/mL)	9.53 (7.24)	8.70 (3.92)	0.245
DHEA (nmol/L)	2085 (1045)	2303 (1038)	0.088
Androstenedione (ng/mL)	1.00 (0.46)	1.14 (0.60)	0.042*
Estradiol (pg/mL)	31.7 (12.2)	34.8 (13.7)	0.051
17-hydroxyprogesterone (ng/mL)	1.00 (0.89)	0.89 (0.63)	0.229
Progesterone (ng/mL)	0.19 (0.09)	0.20 (0.19)	0.496
LH (mUI/mL)	4.12 (2.50)	3.61 (1.63)	0.050

25(OH)D sufficiency and deficiency groups are divided under the 50^th^ percentile of 25(OH)D. Data is expressed as mean ± standard deviations. Asterisk indicates significant difference between 25(OH)D sufficiency and deficiency groups, according to Mann Whitney U test (*p < 0.05).

25(OH)D, 25-hydroxyvitamin D; DHEA, Dehydroepiandrosterone; FSH, Follicle stimulating hormone; IA, Immuno-assay; LC-MS/MS, Liquid chromatography-mass spectrometry/mass spectrometry; LH, Lutein hormone; SHBG, Sex hormone binding globulin.

### Association between sex hormones and serum 25-hydroxyvitamin D through body mass index

We used multivariable lineal regression to investigate the strength of the associations between serum 25(OH)D and testosterone levels shown **Table 2**. As observed in **Table 3**, in model 1, which was unadjusted, we found that 25(OH)D was correlated with the majority of sex hormones, such as total testosterone, SHGB, prolactin, and androstenedione and estradiol. In model 2, which was adjusted by age and smoking status, the previous correlation profile was maintained. Total testosterone, however, was not associated with 25(OH)D, SHGB or estradiol in model 3, which was adjusted by age, smoking status and BMI. Only prolactin and androstenedione remained significantly associated with 25(OH)D, suggesting that BMI had no effect on these associations.

**Table 3:** Linear regression models assessing the association between 25-hydroxyvitamin D with sex-related hormones in men with obesity.

Variables	Model 1β value (SE)	Model 2β value (SE)	Model 3β value (SE)
Total testosterone (ng/mL)	0.932 (0.365)*	0.938 (0.409)*	0.406 (0.432)
Free testosterone (ng/mL)	0.026 (0.017)	0.028 (0.019)	-0.000 (0.020)
SHBG (nmol/L)	0.090 (0.041)*	0.089 (0.045)*	0.072 (0.044)
FSH (pg/mL)	0.355 (0.207)	0.324 (0.226)	0.302 (0.220)
Prolactin (ng/mL)	0.206 (0.089)*	0.239 (0.097)*	0.217 (0.094)*
DHEA (nmol/L)	-0.000 (0.000)	-0.000 (0.000)	-0.000 (0.000)
Androstenedione (ng/mL)	-2.376 (0.950)*	-29.53 (1.054)*	-3.375 (1.022)*
Estradiol (pg/mL)	-0.083 (0.039)*	-0.091 (0.043)*	-0.052 (0.043)
17-OH-hydroxyprogesterone (ng/mL)	0.225 (0.677)	0.312 (0.705)	-0.200 ((0.697)
Progesterone (ng/mL)	-6.162 (3.452)	-5.852 (3.589)	-6.304 (3.483)
LH (mUI/mL)	0.459 (0.244)	0.403 (0.257)	0.322 (0.240)

Model 1 was unadjusted.

Model 2 included age and smoking status.

Model 3 included age, smoking status and body mass index.

Data is expressed as β coefficient (SE). Asterisk indicates significant linear regression (*p < 0.05).

DHEA, Dehydroepiandrosteron; FSH, Follicle stimulating hormone; LH, Lutein hormone; SE, Standard error; SHBG, Sex hormone binding globulin.

Using a mediation analysis, we analyzed how BMI affected the relationship between 25(OH)D and testosterone. We found in this model that 25(OH)D was negatively associated with BMI (a: -0.19 (0.05), *p*<0.05). In addition, BMI was negatively associated with both total and free testosterone levels [bTT (total testosterone): - 0.07 (0.01), *p*<0.05; bFT (free testosterone): -1.54 (0.26), *p*<0.05]. Furthermore, the direct effect of 25(OH)D on total testosterone was significant by removing the effect of the BMI [cTT: 0.03 (0.01), *p*<0.05; but non-significant in the case of free testosterone; cFT: 0.34 (0.22), *p*=0.125]. However, the total effect of 25(OH)D on total testosterone through BMI [c’TT: 0.47 (0.38), *p* = 0.22] was not significant, revealing a total mediation of BMI on the association between 25(OH)D and total testosterone (**Supplementary Figure S1**).

We then investigated the effect of the BMI on the relationship between 25(OH)D and testosterone, by examining different grades of obesity. In this insight, we categorized our population study into three grades of obesity, being obesity type I (BMI ≥30 and <35 kg/m^2^), type II (BMI ≥35 and <40 kg/m^2^) and type III (BMI ≥40 kg/m^2^). All models were adjusted by age and smoking status. In model 1, which includes obesity type I, we found that both total and free testosterone were not associated with 25(OH)D. However, androstenedione and estradiol were negatively associated with 25(OH)D (**Table 4**). We did not find an association between sex hormones and 25(OH)D in model 2, which included obesity type II (**Table 4**). Furthermore, in model 3, we found that total testosterone and SHBG were positively associated with 25(OH)D in participants with obesity type III (**Table 4**). Following this observation, we used a mediation analysis to examine the effect of BMI exerted on the relationship between 25(OH)D and testosterone, only in participants with obesity type III. We found in this model that the 25(OH)D was not associated with BMI [a: -0.06 (0.07), *p*=0.392]. In addition, BMI was negatively associated with total testosterone levels [b: - 0.09 (0.02), *p*<0.05]. Furthermore, we found that the direct effect of 25(OH)D on total testosterone was significant after removing the effect of the BMI [c: 0.05 (0.02), *p*<0.05]. Moreover, the total effect of 25(OH)D on total testosterone through BMI was also significant [c’ = 0.04 (0.02), *p*<0.05], indicating a partial mediation of BMI on the association between 25(OH)D and total testosterone (**Supplementary Figure S2**). Finally, there was no interaction between 25(OH)D and BMI in the relationship with total testosterone levels.

**Table 4:** Linear regression model assessing the association between 25-hydroxyvitamin D with sex-related hormones, analyzing different grades of obesity.

Variables	Model 1β value (SE) Obesity type I	Model 2β value (SE) Obesity type II	Model 3β value (SE) Obesity type III
Total Testosterone (ng/mL)	-0.732 (0.943)	0.258 (0.632)	1.688 (0.585)*
Free Testosterone (ng/mL)	-0.048 (0.042)	0.023 (0.026)	0.035 (0.036)
SHBG (nmol/L)	0.009 (0.100)	-0.038 (0.076)	0.193 (0.054)*
FSH (pg/mL)	0.450 (0.512)	0.058 (0.318)	0.387 (0.330)
Prolactin (ng/mL)	0.206 (0.145)	0.290 (0.193)	0.115 (0.205)
DHEA (nmol/L)	-0.001 (0.001)	0.000 (0.001)	-0.000 (0.000)
Androstenedione (ng/mL)	-4.413 (2.166)*	-2.158 (1.632)	-2.386 (1.521)
Estradiol (pg/mL)	-0.224 (0.097)*	0.124 (0.066)	-0.058 (0.058)
17-OH-hydroxyprogesterone (ng/mL)	-0.330 (2.215)	-0.046 (0.678)	1.780 (2.414)
Progesterone (ng/mL)	-13.397 (9.379)	-3.364 (3.525)	-14.888 (12.223)
LH (mUI/mL)	-0.120 (0.610)	0.253 (0.289)	0.967 (0.509)

Model 1 included age and smoking status. This model included patients with obesity type I (body mass index ≥ 30 and < 35 kg/m^2^; N = 89).

Model 2 included age and smoking status. This model included patients with obesity type II (body mass index ≥ 35 and < 40 kg/m^2^; N = 85).

Model 3 included age and smoking status. This model included patients with obesity type III (body mass index ≥ 40 kg/m^2^; N = 96).

Data is expressed as β coefficient ± SE. Asterisk indicates significant linear Pearson regression (*p < 0.05).

DHEA, Dehydroepiandrosterone; FSH, Follicle stimulating hormone; LH, Lutein hormone; SE, Standard error; SHBG, Sex hormone binding globulin.

## Discussion

In our population of young men with obesity, we found that 25(OH)D was positively associated with total testosterone and other sex hormones, in which BMI mediated this association. We observed that the direct effect of 25(OH)D on the majority of sex hormones disappeared and only indirect effect *via* BMI remained, except for androstenedione and prolactin, indicating a total mediation of BMI in the association between 25(OH)D. However, by categorizing obesity in different grades, we found that serum 25(OH)D was significantly associated with total testosterone levels in only those subjects with morbid obesity, and BMI only exerted a partial mediation on this association. To the best of our knowledge, this is the largest clinical investigation of the relationship between 25(OH)D and testosterone levels in men with obesity, demonstrating a novel effect of BMI on this relationship.

In recent years, hypovitaminosis of vitamin D has been incredibly common in our population, despite vitamin D deficiency having been independently associated with various chronic conditions (21). There is currently controversial statement regarding the best serum concentration of 25(OH)D associated with overall health, (neither for low testosterone levels nor hypogonadism risk). According to the U.S. Institute of Medicine (IOM) expert committee, levels of 20 ng/mL (≥50 nmol/L) or more are optimal for most people, in terms of bone health (22), whereas the Endocrine Society defines that vitamin D sufficiency as an optimal serum 25(OH) equal or greater than 30 ng/mL (23).

The 50^th^ percentile of 25(OH)D in our study was 20 ng/mL (20.10 ng/mL), which is similar to the statement of IOM (22). However, all the participants in our study were subjects with obesity. Therefore, this observation should be considered with caution, and extrapolation of these observations to other populations may not be valid because all participants were obese. In fact, obesity significantly influences circulating levels of vitamin D.

In several epidemiological studies, low levels of vitamin D deficiency have been observed in subjects with obesity (21). Indeed, there are four suggested hypotheses that are mostly accepted within the literature, explaining the vitamin D deficiency status in obesity [reviewed by Pourshahidi L. (2014)] (24). The most accepted mechanism is the volumetric dilution of serum 25(OH)D, in which it is distributed into a larger volume of organs, such as adipose tissue, liver, muscle, etc., taking a greater serum 25(OH)D from the bloodstream (4). However, despite the high prevalence of vitamin D deficiency in subjects with obesity, this condition does not seem to have a greater impact on bone mineral density and bone metabolism (25), but may be implicated in other systemic consequences and chronic diseases (26), such as decreased testosterone levels and an increased hypogonadism risk (27). Because vitamin D is important in body fat, insulin sensitivity, adipogenesis and lipid accumulation, as well as cytokine and inflammatory signaling in adipose tissue, the health consequence of low serum 25(OH)D on adipose metabolic functions in obesity is highly documented (4). As a result, low serum 25(OH)D status may impair the well-functioning of adipose tissue, and increase the risk of several local-metabolic disorders, such as low-grade inflammatory state, or insulin resistance.

On the other hand, testosterone deficiency is a very common feature in males with obesity, in which there is a causal effect of an increased BMI on serum testosterone levels in men (28). That is, because fat cells in moderate obesity predominantly metabolize testosterone to estrogens and consequently decrease their serum levels. Increased estrogen production, therefore, has been associated with negative feedback on LH secretion (29). The presence of moderate obesity also causes reductions in total testosterone levels, as a consequence of reductions in SHBG, due to insulin resistance-associated mechanisms and obesity-associated hyperinsulinemia (30).

Testosterone deficiency was also related to serum vitamin D. The most recent systematic review and meta-analysis, conducted by D’Andrea et al. (2021), which included 18 studies (9,892 men with vitamin D deficiency and 10,675 controls), revealed a slight, but positive association between 25(OH)D and total testosterone levels (14), which we also found in our study. We noted that both free and total testosterone levels are found to be decreased in subjects with vitamin D deficiency. However, additional studies did not support this observation (31), and vitamin D supplementation was not able to increase serum testosterone levels (32, 33). In the linear regression analysis, we found in an unadjusted model that 25(OH)D was associated with total testosterone levels. However, this association disappeared by adjusting age, smoking and BMI, indicating a total mediation effect of BMI on this association. Indeed, a refined analysis revealed that the association between 25(OH)D and total testosterone levels is only significant in participants with morbid obesity. This observation suggests that vitamin D may have a positive effect on low testosterone levels, as observed in morbid obesity, rather than slightly reduced testosterone levels. As a result, these results could suggest that vitamin D supplementation may be more beneficial if it is targeted to those subjects with very low serum testosterone levels, since many studies have failed to show an increase in testosterone with vitamin D supplementation (32, 34, 35).

We also observed in our study that subjects with vitamin D deficiency had increased androstenedione when compared with participants with vitamin D sufficiency. Accordingly, an *in vitro* study showed that treatment of human adrenocortical cells with 1α,25-Dihydroxyvitamin D_3_ decreased androstenedione secretion, by affecting steroidogenic enzymes (36). However, literature about vitamin D and androstenedione is scarce. Some studies in vitamin-D-deficient polycystic ovary syndrome patients after vitamin D supplementation, showed that serum androstenedione levels had significantly decreased androstenedione levels (37), although other studies did not confirm this observation (38). After controlling for age, smoking status, and BMI, we discovered that 25(OH)D was still associated with androstenedione in our linear regression analysis. Indeed, this association was only observed in those subjects that had obesity type I, suggesting that this relationship between 25(OH)D and androstenedione has not fully been influenced by BMI (39). Therefore, further studies are needed to elucidate this mechanism (34, 35).

Our study has various strengths, that are worth mentioning. First, this is the first study to examine the effect of BMI on the relationship between 25(OH)D and testosterone, adding new evidence to the body of knowledge about the impact of BMI on metabolic hormones. Another strength of our study is that testosterone levels were measured by HPLC, providing a more precise value. In addition, the recruitment of the participants, as they all proceeded from primary care, avoided a selection bias with specialty-care patients, which could potentially have more comorbidities related to hypovitaminosis D or low testosterone levels. However, our study had several limitations to point out. First, the cross-sectional nature of this study may be one of these limitations, as we can only hypothesize based on the cross-sectional design. Furthermore, the participants are recruited in the same geographic region. They may not represent the Spanish general population, and we cannot establish causal relations among variables. Second, due to the strategy used to recruit the participants for this study, we omitted the seasonal variation analysis, despite the fact that 25(OH)D may vary depending on the seasons, although a preliminary analysis found no differences on seasonal variation. Another limitation of this study is the small size. Although the sample size used seemed to be relatively small, our recruited model was based on cross-sectional nature and restricted inclusion and exclusion criteria, which it has limited the number of participants. Finally, the effects of additional factors may also alter the status of circulating vitamin D. Some factors, such as genetics, microbiota, aging, or physical activity, have been demonstrated to exert a potential effect on the status of vitamin D. However, the authors adjusted the models by those controlling variables, such as smoking status or age, to limit the effect of these cofounding variables.

## Conclusion

We observed a mediation of BMI on the association between 25(OH)D and sex hormones. Except for the androstenedione and prolactin, the direct effect of 25(OH)D on the majority of sex hormones disappeared and only indirect effect *via* BMI remained. However, when different grades of obesity were examined, serum 25(OH)D levels were found to be related to total testosterone and SHBG levels in participants with morbid obesity, suggesting that vitamin D may have specific benefits in those subjects with very low testosterone levels and severe cases of obesity. Additional research is needed to elucidate possible common mechanisms.

## Data availability statement

The raw data supporting the conclusions of this article will be made available by the authors, without undue reservation.

## Ethics statement

The Ethics and Research Committee of the Hospital Clínico Virgen de la Victoria approved the experimental design. The participants gave their written informed consent to participate in this study.

## Author contributions

Conceptualization: JF-G, MD-F, MM-G, HB and FT. Data curation: HB, MD-F, MM-G. Formal analysis: HB, MM-G, MD-F. Funding acquisition: JF-G, FT. Investigation: JF-G, MM-V, MD-F, FT. Methodology: MM-V, MD-F, HB, MM-G, FT. Project administration: JF-G, FT, MM-G. Writing – original draft: HB, MM-G, MD-F. Writing – review and editing: All authors. All authors contributed to the article and approved the submitted version.

## Funding

The research group belongs to the “Centros de Investigacion en Red” [CIBERobn, of the “Instituto de Salud Carlos III]. JCF-G was supported by an intensification research program (INT21/00078, ISCIII, Spain; co-funded by the Fondo Europeo de Desarrollo Regional-FEDER); This work was supported in part by a grant from Servicio Andaluz de Salud (PI-0173-2013). MMG. was the recipient of the Nicolas Monardes Program from the “Servicio Andaluz de Salud, Junta de Andaluciía”, Spain (RC-0001-2018 and C-0029-2014). HB is supported by a predoctoral fellowship (“Plan Propio IBIMA 2020 A.1 Contratos predoctorales”, Ref.: predoc20_002). MDF was supported by a Rio Hortega grant from Instituto de Salud Carlos III, Madrid, Spain (CM20/00183). The funders had no role in study design, data collection and analysis, decision to publish, or preparation of the manuscript

## Acknowledgments

We would like to thank all the participants of this research.

## Conflict of interest

The authors declare that the research was conducted in the absence of any commercial or financial relationships that could be construed as a potential conflict of interest.

## Publisher’s note

All claims expressed in this article are solely those of the authors and do not necessarily represent those of their affiliated organizations, or those of the publisher, the editors and the reviewers. Any product that may be evaluated in this article, or claim that may be made by its manufacturer, is not guaranteed or endorsed by the publisher.
