# Supplementary material for: 25-hydroxyvitamin D and testosterone levels association through body mass index: A cross-sectional study of young men with obesity
Source: Front Endocrinol (Lausanne). 2022 Sep 2;13:960222. doi: 10.3389/fendo.2022.960222 (PMC9478588; doi:10.3389/fendo.2022.960222)
Supplement: Supplementary file 1 [file DataSheet_1.pdf]

Supplementary Figure 1

Causal mediation model of the effect of 25-hydroxyvitamin D and testosterone levels through body mass index.

Asterisks indicate significant p-values (*p<0.05). Significance of the indirect effect was determined using mean bootstrapped.

- a: Direct effect of 25(OH)D on testosterone levels;
- b: Direct effect of 25(OH)D on body mass index;
- c: Direct effect of 25(OH)D on testosterone removing body mass index
- c´: Total effect of 25(OH)D on testosterone levels through body mass index;

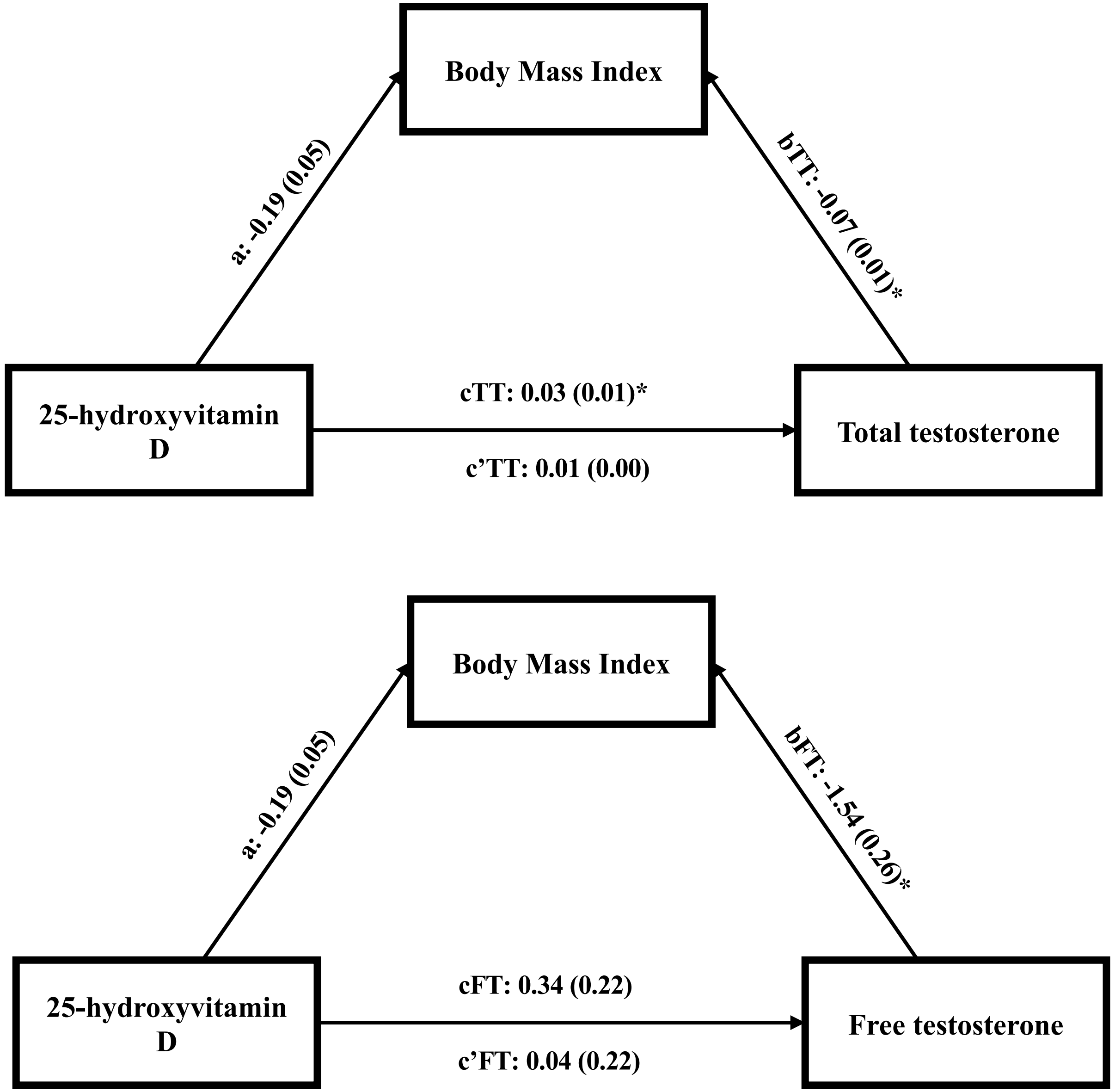
Body Mass Index
a: -0.19 (0.05)
bTT: -0.07 (0.01)*
25-hydroxyvitamin D
cTT: 0.03 (0.01)*
c’TT: 0.01 (0.00)
Total testosterone
Body Mass Index
a: -0.19 (0.05)
bFT: -1.54 (0.26)*
25-hydroxyvitamin D
cFT: 0.34 (0.22)
c’FT: 0.04 (0.22)
Free testosterone
