# Supplementary material for: 25-hydroxyvitamin D and testosterone levels association through body mass index: A cross-sectional study of young men with obesity
Source: Front Endocrinol (Lausanne). 2022 Sep 2;13:960222. doi: 10.3389/fendo.2022.960222 (PMC9478588; doi:10.3389/fendo.2022.960222)
Supplement: Supplementary file 2 [file DataSheet_2.pdf]

Supplementary Figure 2

Causal mediation model of the effect of 25-hydroxyvitamin D and testosterone levels through body mass index, in patients with morbid obesity.

Asterisks indicate significant p-values (*$p<0.05$). Significance of the indirect effect was determined using mean bootstrapped.

- a: Direct effect of 25(OH)D on testosterone levels;
- b: Direct effect of 25(OH)D on body mass index;
- c: Direct effect of 25(OH)D on testosterone removing body mass index
- c´: Total effect of 25(OH)D on testosterone levels through body mass index;

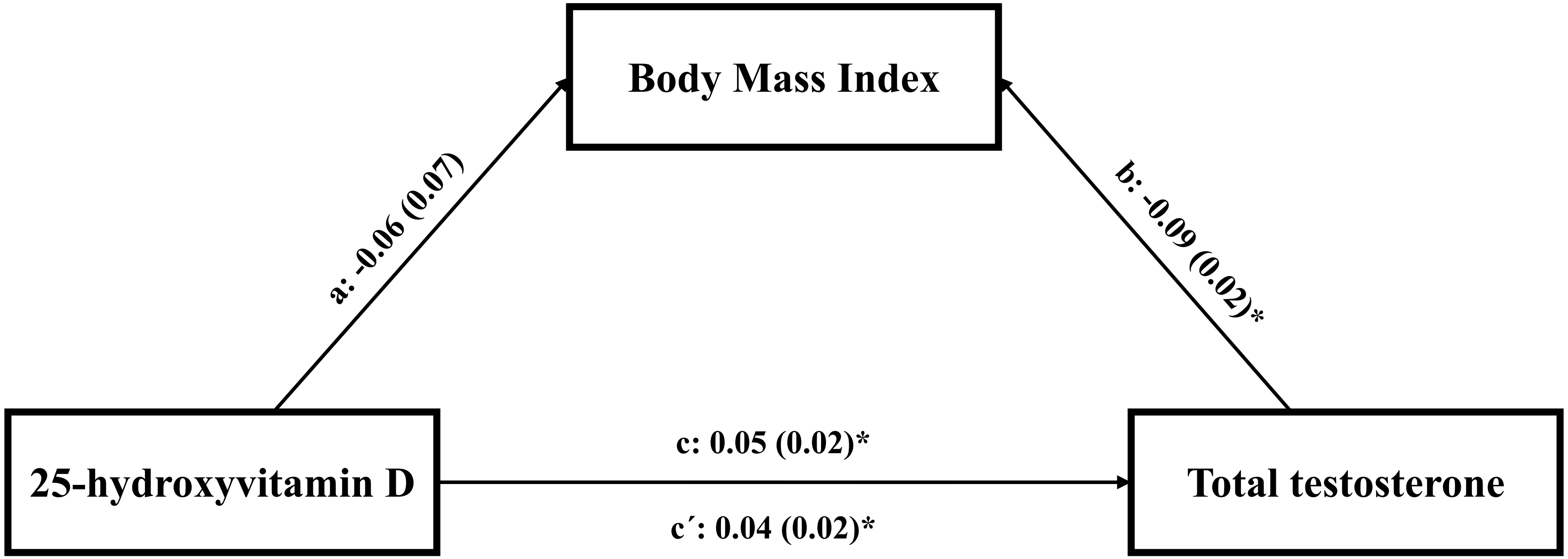
Body Mass Index
a: -0.06 (0.07)
b: -0.09 (0.02)*
25-hydroxyvitamin D
c: 0.05 (0.02)*
c´: 0.04 (0.02)*
Total testosterone
